# “Universal test and treat” program reduced TB incidence by 75% among a cohort of adults taking antiretroviral therapy (ART) in Gurage zone, South Ethiopia

**DOI:** 10.1186/s40794-020-00113-3

**Published:** 2020-07-31

**Authors:** Tadele Girum, Fedila Yasin, Samuel Dessu, Bereket Zeleke, Mulugeta Geremew

**Affiliations:** 1 Department of Public health, College of Medicine and Health Sciences, Wolkite University, Wolkite, Ethiopia; 2 Department of Pharmacy, College of Medicine and Health Sciences, Wolkite University, Wolkite, Ethiopia; 3 Department of Statistics, College of Computing and informatics, Wolkite University, Wolkite, Ethiopia

**Keywords:** Universal test and treat, Differed treatment, Tuberculosis, Incidence

## Abstract

**Background:**

Tuberculosis (TB) remains the leading cause of morbidity and mortality in peoples living with HIV and at least 25% of deaths are attributed to TB. Many countries implement the Universal Test and Treat (UTT) program for HIV, which is believed to reduce the incidence of TB. However, there are limited studies that evaluate the impact of UTT on TB incidence. Therefore, by recruiting a cohort of ART users in the “UTT” and “differed treatment” programs, we aim to measure the effect of the UTT program on TB incidence.

**Objective:**

To measure the effect of “UTT” program on TB incidence among a cohort of adults taking antiretroviral therapy (ART) in Gurage Zone, South Ethiopia.

**Methods:**

A retrospective cohort study was conducted through record review over 5 years (2014–2019) in public health facilities in Gurage Zone. Three hundred eighty-four records were randomly selected and reviewed using a standardized structured checklist. Data was entered using Epi Info™ Version 7 and analyzed by STATA. A generalized linear model with binomial link function was fitted to measure the adjusted incidence density/incidence rate ratio and to identify predictors of incidence difference between the two programs.

**Results:**

During the follow up period, 39 incident TB cases were identified with an overall incidence rate of 4.79/100 person-year (PY). TB incidence was significantly lower in the UTT cohort (IR = 2.10/100 PY) in comparison to the differed program cohort (IR = 6.23/100 PY). The adjusted incidence rate ratio (AIRR) of TB among patients enrolled in the UTT program was; 0.25 (95% CI = 0.08–0.70). Thus, there was a reduction of TB incidence by 75% in the UTT program compared to differed program. In addition, IPT (isoniazid preventive therapy) use (AIRR = 0.35 (95% CI = 0.22–0.48)), WHO Stage I and II (AIRR = 0.70 (95% CI = 0.61–0.94)) and higher base line CD4 count (AIRR = 0.96 (95% CI = .94–0.99)) significantly reduced the incidence of TB. However, treatment failure increase the incidence (AIRR = 5.8 (95% CI = 1.93–8.46)).

**Conclusion:**

TB incidence was significantly reduced by 75% after UTT. Therefore, intervention to further reduce the incidence has to focus on strengthening UTT program and IPT.

## Background

Tuberculosis (TB) is a major global public health problem [1, 2]. It is the ninth leading cause of death worldwide and the leading cause from a single infectious agent [2, 3]. In 2018, 10 million new cases of TB occurred, of which 8.6% were among people living with HIV. Thus, the risk of developing active TB in people living with HIV (PLWH) is 19 times higher than in the general population [2, 4, 5]. In the same year, TB infection caused 1.45 million deaths [2], including 251,000 deaths among HIV positive people. The African region accounts the largest share of these deaths [5–7].

The risk of developing TB increase as the CD4 count decreases and altered immune status [7–9]. At least 25% of deaths among PLWHA are attributed to TB and many of these deaths occur in developing African countries [10]. HIV positive people with latent TB infection have a 10% annual and 50% lifetime risk of developing active TB disease, compared to a 10% life time risk among HIV negative individuals [10, 11]. In areas where the HIV infection are prevalent, TB continues to be a major public health problem [1–3].

HIV and TB management programs are provided in integrated approaches at clinical and public health system level [1, 2]. The differed program used CD4 count and/or WHO clinical stage as criteria for initiating HIV treatment. In comparison, the UTT recommends initiation of HIV treatment regardless of CD4 level and WHO clinical stage [1, 5, 10]. Thus, UTT facilitates early initiation of treatment and has an impact on TB epidemiology, particularly in reducing the incidence of active TB [1, 2, 12].

Previous studies reported that ART reduces the incidence of TB [13–18]. One of these studies reported that the risk of TB is reduced by 65% through ART initiation [13]. ART also reduced mortality from TB by 95% [18]. Other studies have also consistently shown the benefit of ART on TB outcomes, particularly when ART is initiated early [17–20]. One observational study reported that early initiation of ART in HIV-infected TB patients reduced TB incidence rates by 90% at an individual level [18] and by 60% at a population level [19]. ART also has been shown to reduce TB recurrence rates by 50% [20].

Ethiopia is one of the SSA countries with the highest prevalence of TB/HIV co-infection and ranked seventh among the world’s 30 highest TB burden countries [6]. In 2017, according to the Centers for Diseases Control and Prevention (CDC), the incidence rate of TB in Ethiopia was 164 cases per 100,000 population including approximately 7% who were PLHIV. In the same year, the mortality rate of TB patients in Ethiopia was 24 per 100,000 [2, 21].

Ethiopia has started the UTT program in 2016 in order to alter the epidemiology of HIV infection. It is believed that, the program facilitates early initiation of ART and subsequently reduces incidence of TB and other opportunistic infections [2]. However, there is no study conducted to evaluate the impact of UTT on incidence of TB. Therefore, by recruiting a cohort of ART users in the new (UTT) and previous (differed treatment) programs we aimed to assess the effect of UTT program in incidence of TB among PLWHA. The evidence will be used as base line information for planners, implementers and organizations.

## Methods and materials

### Study design and settings

A retrospective cohort study was conducted in ART clinics found in Gurage Zone, Southern Ethiopia between May and June 2019 by reviewing outcomes over 5 years (2014–2019). Gurage Zone is one of the 13 zones available in SNNPRs. Wolkite is the capital of the zone located 158 km south of the capital city Addis Ababa. The zone has 16 districts and 5 town administrations. There are 76 health centers and 6 hospitals. Of these, 20 health facilities provide HIV care and treatment in the area.

### Study population, sample size and sampling technique

The study population were all adults aged > 15 years with an HIV positive diagnosis and enrolled in a treatment program in ART clinics found in Gurage Zone. Of the 20 facilities, 5 facilities were randomly selected and the patients were proportionally allocated to the facilities based on patient load. The sample size was calculated based on a double population proportion formula by using Epi Info™ Version 7 computer program with the following assumptions: a 0.5 risk ratio within 95% confidence level, power of 80%, ratio of unexposed to exposed 2:1 and an outcome in un-exposed =28.9% [22]. Twenty percent was added to account for incompleteness, resulting in a sample size of 392 (131 exposed and 261 unexposed).

### Data collection procedure and data quality control

The Pre-ART register, ART register, patients’ ART follow up and medical charts were sources of data. In these registers and follow up charts, clients’ sociodemographics, clinical and laboratory information, treatments, the follow-up status of each client, and incidence of TB were recorded. Data was collected using a structured checklist for records review developed from the registers and follow up charts. Five data collectors and three supervisors working in ART clinics were recruited for data collection.

### Study variables

The outcome measured was the incidence of TB after enrolment with in the HAART program. It was measured as the number of incident cases per person year of follow-up.

The two groups (exposed and unexposed) were determined based on the enrolled program (UTT Vs differed). Theoutcome (development of TB) was ascertained by 4 criteria according to the national guideline. 1. sputum smear test, 2. chest X-Ray, 3. culture and 4. clinical (physician) judgment (in the case where other tests were reported negative and sign and symptoms clearly indicated the presence of TB infection). Both the exposure status and outcome status were retrospectively reviewed from records.

### Data processing and analysis

The data was entered into Epi-Info™ Version 7 and exported to STATA version 11 for statistical analysis. After exploratory data analyses and assumption tests, a generalized linear model with binomial link function was fitted to measure adjusted incidence density and identify predictors of incidence difference. Crude and adjusted incidence rate ratio were measured and reported with a 95% confidence interval.

## Results

### Socio-demographic characteristics

A total of 392 randomly selected patients’ charts were reviewed with structured check list and eight were found to be incomplete. Out of the final 384 records that were fully reviewed, 128 patients (33.3%) were enrolled in the UTT program. Nearly two-third (68.7%) of the patients enrolled into the study were females and 214 (55.7%) were urban residents. Half (51%) of the clients were married and more than one third (37%) of patients has no formal education. Regarding their occupational status, 30% of them were unemployed. The mean age at the time of diagnosis was 34.8 years (SD = 9.1) with no significant difference between the two programs (Table 1).

**Table 1:** Socio-demographic characteristics of HIV infected patients enrolled in UTT and differed programs in Gurage Zone, 2019

Variables	Outcome by program				
	UTT (N, %)		CD4 based (N, %)		Total
	TB	NO TB	TB	NO TB	(N, %)
Mean age at DX	34.7+ 8.8		35.1+ 9.2		34.8+ 9.1
Sex					
Male	2 (33.3)	47 (38.5)	12 (36.4)	59 (26.5)	120 (31.2)
Female	4 (66.7)	75 (61.5)	21 (63.6)	164 (73.5)	264 (68.7)
Residence					
Rural	4 (66.7)	38 (31)	22 (66.6)	106 (47.5)	170 (44.3)
Urban	2 (33.3)	84 (69)	11 (33.4)	117 (52.5)	214 (55.7)
Marital status					
Single	1 (16.7)	17 (14)	5 (15)	40 (18)	63 (16.4)
Married	2 (33.3)	69 (56.5)	21 (63.6)	104 (46.6)	196 (51)
Divorced	3 (50)	36 (29.5)	7 (21.4)	79 (35.4)	125 (32.6)
Educational status					
Illiterate	4 (66.7)	44 (36)	12 (36.4)	82 (36.8)	142 (37)
Literate	2 (33.3)	78 (64)	21 (63.6)	141 (63.2)	242 (63)
Employment status					
Employed	3 (50)	92 (75.4)	18 (54.5)	156 (70)	269 (70)
Unemployed	3 (50)	30 (24.6)	15 (45.5)	67 (30)	115 (30)

*UTT* Universal test and treat, *DX* Diagnosis, *TB* Tuberculosis

### Baseline and clinical characteristics

The median time from diagnosis to initiation of treatment was 0.7 years (IQR = 0.2–1.2). The average weight of participants was 52.3 kg (SD = 11.3 kg) and; patients in the UTT program had a slightly higher weight (52.5+ 2 Kg) than patients in the differed program (51 + 9 kg). The median CD4 count up on initiation of ART was 201 (IQR: 126–303), which was higher among patients in the UTT program 262.4 (IQR: 130–568) than the differed treatment (181, IQR: 110–235) (Table 2).

**Table 2:** Baseline clinical characteristics of HIV infected patients enrolled in UTT and differed programs in Gurage Zone, 2019

Variables	Outcome by Program				
	UTT (N, %)		CD4 based (N, %)		Total
	TB	NO TB	TB	NO TB	(N, %)
WHO stage					
Stage I	0 (0)	38 (31.1)	3 (9.1)	48 (21.5)	89 (23.1)
Stage II	1 (16.7)	38 (31.1)	5 (15.3)	53 (23.8)	97 (25.2)
Stage III	3 (50)	40 (32.8)	22 (66.7)	115 (51.5)	180 (47)
Stage IV	2 (33.3)	6 (4.9)	3 (9)	7 (3.2)	18 (4.7)
Median CD4 count	262.4 (IQR: 130–568)		181 (IQR: 110–235)		201 (IQR: 126–303)
Mean weight	52.5+ 2		51 + 9		52.3+ 11.3
Median time to Rx	0.3 (IQR = 0.1–4.2)		0.9 (IQR = 0.6–1.6)		0.7 (IQR = 0.2–1.2)
OIS before ART					
Yes	3 (50)	49 (40.2)	22 (66.7)	116 (52)	190 (49.5)
No	3 (50)	73 (59.8)	11 (33.3)	107 (48)	194 (50.5)
IPT					
Yes	4 (66.7)	113 (92.6)	11 (33.3)	155 (69.5)	283 (73.7)
No	2 (33.3)	9 (7.4)	22 (66.7)	68 (30.5)	101 (26.3)
Treatment failure					
Yes	3 (50)	9 (7.4)	2 (6)	6 (2.7)	20 (5.2)
No	3 (50)	113 (92.6)	31 (94)	217 (97.3)	364 (94.8)

*IPT* Isoniazid preventive therapy, *RX* Treatment

During the initiation of ART 51.7% of the patients were in WHO clinical stage III and IV in both groups. In the UTT program, only 39.8% of patients were in WHO clinical stage III and IV, whereas in the deferred treatment program nearly 57.4% of patients were in WHO clinical stage III and IV. More than two-third of patients received IPT (isoniazid preventive therapy- a prophylaxis which is given to prevent active TB). Half of the patients were diagnosed to have at least one opportunistic infection before ART initiation. 5.2% of patients developed treatment failure in the course of ART treatment (Table 2).

### Incidence rate of TB

Among the 384 HIV-infected patients who were followed for a total of 9766 person-month, 39 incident TB cases were identified, making the overall incidence of TB 4.79/100 PY. The overall incidence density rate (IDR) of TB was significantly different for the two comparison groups. The incidence was 2.10/100 PY in the UTT and 6.23/100 PY in the differed treatment program with a p.value of 0.003. After adjusting for the effect of other variables the adjusted incidence rate Ratio (AIRR) of TB among patients enrolled in the UTT program compared to patients enrolled in the differed program was; 0.25(0.08–0.70). Thus, the UTT program reduced TB incidence by 75% compared to the differed treatment program (Table 3).

**Table 3:** Univariate and multivariate analysis of Incidence rate of TB among clients enrolled in UTT and differed programs in Gurage Zone, 2019

Predictors	Event/p-M	IR/100P-Y	CIRR(95%CI)	*p*.value	AIRR(95%CI)
Over all	39/9766	4.79	–	–	–
Program					
UTT	6/ 3417	2.10	0.33 (0.11–0.81)	0.003	0.25 (0.08–0.70)*
Differed	33/6349	6.23	1		1
Age	–	–	1.01 (0.97–1.04)	0.622	–
Sex					
Male	14/3134	5.36	1.18 (0.56–2.37)	0.230	1.33 (0.64–2.75)
Female	25/6632	4.52	1		1
Residence					
Rural	26/ 4094	7.6	2.77 (1.42–5.38)	0.001	1.58 (0.82–3.05)
Urban	13/5672	2.75	1		
Employment					
Employed	21/6999	3.60	1		1
Unemployed	18/2767	7.80	2.16 (1.34–5.10)	0.001	2.2 (0.82–4.54)
Marital status					
Married	23/4920	5.60	1.41 (0.71–2.86)	0.14	1.73 (0.88–3.40)
Unmarried	16/4846	3.96	1		1
Educational status					
Illiterate	16/3680	5.22	1.15 (0.56–2.27)	0.33	
Literate	23/ 6086	4.53	1		
Weight	–	–	0.97 (0.94–1.00)	0.110	0.97 (0.94–1.01)
WHO stage					
Stage I/II	9/3600	3.00	0.51 (0.26–0.96)	0.018	0.70 (0.61–0.94)*
Stage III/IV	30/6166	5.84	1		1
IPT					
Yes	15/6800	2.64	0.27 (0.18–0.56)	0.001	0.35 (0.22–0.48)*
No	24/2966	9.71	1		1
Treatment failure					
Yes	5/ 422	14.2	3.25 (1.10–8.36)	0.015	5.80 (1.93–8.46)*
No	34/ 9344	4.37	1		1
Base line CD4 count	–	–	0.99 (0.98–1.00)	0.050	0.96(.94–0.99)*

*AIRR Adjusted Incidence Rate Ratio, *IRR Incidence Rate Ratio, *CIRR Crude Incidence Rate Ratio

Also, the incidence differed in different subgroups. Males have a TB incidence of 5.36/100-person-year compared to 4.52/100-person-year incidence among females. Rural residents have a far greater risk of developing TB with, 7.6/100-PY than urban residents which is 2.75/100-person-year. Also, the incidence of TB was higher for unemployed patients, advanced clinical stage patients, and patients that have experienced treatment failure (Table 3).

### Factors associated with TB incidence among HIV infected patients

Through a bivariate analysis, program of enrolment, residence, employment status, marital status, sex, base line weight, history of IPT, base line CD4 count and treatment failure were associated with TB incidence (*p*.value less than 0.25). A multivariate analysis was conducted on the above variables. After controlling for the effect of other variables program of enrolment, WHO clinical stage, IPT, treatment failure and base line CD4 count were significantly associated with TB incidence among HIV/AIDS infected patients in the course of treatment (Table 3).

After controlling for the effect of other variables, patients enrolled in the UTT program were 75% less likely to develop TB than patients enrolled in the differed program with, AIRR = 0.25 (95% CI [0.08–0.70], *p*.value < 0.001). Additionally, patients who were in WHO clinical stage one and two were 30% less likely to develop TB than patients in clinical stage three and four with; AIRR = 0.70 (95% CI [0.61–0.94], *p*.value < 0.001). Meanwhile, having IPT reduces the risk of developing TB by two-thirds; AIRR = 0.35 (95% CI [0.22–0.48], *p*.value < 0.001). The Risk of developing TB was 5.80 (95% CI [1.93–8.46], *p*.value < 0.001) times higher for patients who have treatment failure than those that didn’t have failure history. An increase of base-line CD4 count by one unit reduced the risk of developing TB by 4 % (Table 3).

## Discussion

This retrospective cohort study assessed the effect of the UTT program on TB incidence among a cohort of HIV infected patients enrolled in the HAART program between 2014 and 2019 in public health facilities of Gurage Zone, South Ethiopia. The overall incidence rate (IR) was 4.79/100 PY. The TB incidence found in our study was far lower than previous studies conducted in Hawassa (8.79/100PY) [23] and, Gondar (7.88 per 100PY) [22], and comparison to a meta-analysis conducted in Ethiopia (10.5/100PY) [24]. On the other hand, this finding is slightly lower than a study conducted at the Debre Markos referral hospital in North Ethiopia (6.5 per 100PY) [25]. The observed difference may have been occurred due to time difference in initiation of treatment, overall TB prevention program and service [23–25].

The IR among patients enrolled in the UTT program was lower than patients enrolled in the differed program (2.10/100-Person-Year Vs. 6.23/100-Person-Year). Thus, UTT program significantly reduced TB incidence by 75% when compared to the differed program. This may be as a result of early initiation and strong follow up [19, 24, 25]. In addition to this, patient enrolled in the UTT program were initiated ART at higher CD4 level which helps to prevent TB. Previous research indicated that early initiation of ART in HIV-infected TB patients reduces TB incidence rates by 90% on an individual level [18].

Although, overall incidence and life time risk of TB among HIV infected patients have been reduced in the last few years in high burden countries like Ethiopia, such a remarkable reduction due to the effect of UTT program and overall reduction of HIV epidemics is a promising finding [1, 2, 11, 26]. Expanding and strengthening the program along with early initiation, drug adherence, and the implementation of an overall TB prevention program may has the potential to further enhance the impact of UTT program on reducing TB incidence and reach the 90–90-90 target.

In this study, in addition to the type of program which patients were enrolled, provision of IPT significantly reduced the incidence of TB. Patients who received IPT have a 65% lower risk of developing TB than patients who didn’t received IPT. The results are corroborated by similar outcomes reported in other studies, which found that IPT was preventative in different settings [13, 16, 22, 24, 25, 27]. A pooled estimate in a study conducted in Ethiopia reported that IPT reduces the incidence of active TB among HIV positive patients by 74% [24]. The national guide lines and WHO guide line recommends implementation of IPT as the mainstay managment to reduce incidence of TB among HIV infected patients particularly in high TB/HIV burden countries [10, 11, 27].

Other important factors found to reduce the risk of developing TB among HIV infected patients were WHO clinical stage and base line CD4 count. Patients in WHO clinical stage one and two reduced the incidence of TB by 30% compared to advanced stages (stage three and four). Similarly, a unit increase in base line CD4 count reduced incidence of TB by 4% among HIV infected patients. This is in line with previous studies, where clinical stages and bases line CD4 counts were the most important factors for TB development [22, 23, 25]. This is because, TB develops at advanced stages of HIV when CD4 count are lower and patients are immunecompromised [2, 11, 24, 27].

On the other hand development of treatment failure increased the risk of TB incidence by six-fold. Treatment failure and drug resistance increased viral load and reduced CD4 count. Consequently, it increased the risk of opportunistic infections including TB. It is well-documented in past literature that treatment failure results in multiples adverse outcomes in HIV treatment [1, 9, 11, 22, 23, 25]. Most commonly, it is associated with fatal opportunistic infections like TB [2, 11, 18, 23].

### Strengths and limitations of the study

To the extent of the researchers’ knowledge there is no research that assessed the impact of the UTT program on TB incidence among HIV infected patients since the program was started in Ethiopia. Therefore, this research will bring forth a new evidence on the impact of UTT in clinical setting. The findings of this study might be limited by the fact that it is retrospective study and based on record review. Therefore, incompleteness of information and reliability of the recorded data remains a concern.

## Conclusion and recommendations

This study found that UTT program significantly reduced TB incidence by 75% when compared to the differed program among HIV-infected patients. The overall incidence density rate (IDR) of death in the cohort was lower than other studies that were previously conducted. In addition to the program of enrolment, being in an early WHO clinical stage, having IPT exposure and having higher base line CD4 count significantly reduced the incidence of TB. The risk of developing TB increased as patients develop treatment failure. Therefore, intervention to further reduce TB incidence should focus on strengthening the UTT program to initiate treatment as early as possible and to prevent treatment failure. The findings of this study may provide necessary information on areas of improvement; however, further research is needed to provide policy level recommendations.

## Data Availability

Please contact author for data requests.

## Abbreviations

AIRR: Adjusted Incidence Rate Ratio
ART: Antiretroviral therapy
HAART: Highly Active Antiretroviral therapy
HIV: Human immunodeficiency virus
IDR: Incidence density rate
IPT: Isoniazid preventive therapy
IRR: Incidence Rate Ratio
OIs: Opportunistic infections
TB: Tuberculosis
UTT: Universal test and treat
WHO: World Health Organization
